# Examining Homophily, Language Coordination, and Analytical Thinking in Web-Based Conversations About Vaccines on Reddit: Study Using Deep Neural Network Language Models and Computer-Assisted Conversational Analyses

**DOI:** 10.2196/41882

**Published:** 2023-03-23

**Authors:** Yue Li, William Gee, Kun Jin, Robert Bond

**Affiliations:** 1 School of Communication The Ohio State University Columbus, OH United States; 2 Department of Computer Science and Engineering The Ohio State University Columbus, OH United States

**Keywords:** vaccine hesitancy, social media, web-based conversations, neural network language models, computer-assisted conversational analyses

## Abstract

**Background:**

Vaccine hesitancy has been deemed one of the top 10 threats to global health. Antivaccine information on social media is a major barrier to addressing vaccine hesitancy. Understanding how vaccine proponents and opponents interact with each other on social media may help address vaccine hesitancy.

**Objective:**

We aimed to examine conversations between vaccine proponents and opponents on Reddit to understand whether homophily in web-based conversations impedes opinion exchange, whether people are able to accommodate their languages to each other in web-based conversations, and whether engaging with opposing viewpoints stimulates higher levels of analytical thinking.

**Methods:**

We analyzed large-scale conversational text data about human vaccines on Reddit from 2016 to 2018. Using deep neural network language models and computer-assisted conversational analyses, we obtained each Redditor’s stance on vaccines, each post’s stance on vaccines, each Redditor’s language coordination score, and each post or comment’s analytical thinking score. We then performed chi-square tests, 2-tailed *t* tests, and multilevel modeling to test 3 questions of interest.

**Results:**

The results show that both provaccine and antivaccine Redditors are more likely to selectively respond to Redditors who indicate similar views on vaccines (*P*<.001). When Redditors interact with others who hold opposing views on vaccines, both provaccine and antivaccine Redditors accommodate their language to out-group members (provaccine Redditors: *P*=.044; antivaccine Redditors: *P*=.047) and show no difference in analytical thinking compared with interacting with congruent views (*P*=.63), suggesting that Redditors do not engage in motivated reasoning. Antivaccine Redditors, on average, showed higher analytical thinking in their posts and comments than provaccine Redditors (*P*<.001).

**Conclusions:**

This study shows that although vaccine proponents and opponents selectively communicate with their in-group members on Reddit, they accommodate their language and do not engage in motivated reasoning when communicating with out-group members. These findings may have implications for the design of provaccine campaigns on social media.

## Introduction

### Background

The COVID-19 pandemic thrust vaccine hesitancy that has existed long before the pandemic entered the global spotlight. Vaccine hesitancy, the “delay in acceptance or refusal of vaccine despite of vaccine availability” [1], has been deemed one of the top 10 threats to global health by the World Health Organization. Vaccine hesitancy impedes efforts to treat vaccine-preventable diseases, resulting in millions of unnecessary deaths [1]. Antivaccine groups on social media appear to drive an increase in vaccine hesitancy. As people increasingly consult social media for health information, among whom 16% seek vaccine-related information [2], understanding how people with different attitudes toward vaccines discuss them on social media may help policy makers and practitioners develop strategies to address vaccine hesitancy.

Web-based conversations, such as those about vaccines examined in this study, belong to a broader category of sociable conversations in which individuals discuss everyday interest- or leisure-related issues [3]. Sociable conversations are distinct from formal discussions because the former emphasize casual and spontaneous exchanges of everyday issues, whereas the latter focus on rational exchanges of arguments or opinions [4]. In this study, we defined web-based conversations as the sociable interactions between people about a certain topic (in our case, about vaccines) on social media that typically take the form of interplay between posts and comments. As a specific type of sociable conversation, web-based conversations provide opportunities for the reciprocal exchange of arguments, for reflecting on one’s own thinking, and for building agreement on a variety of issues [5]. In the context of vaccines, vaccine-related conversations on social media may provide an open channel through which people with differing opinions on vaccines may understand each other’s perspectives. Furthermore, web-based conversations may stimulate cognitive information processing [6,7], which is beneficial for building essential knowledge about complex vaccine-related issues.

Although previous research has used different approaches to examine web-based conversations, including their frequency, valence, diffusion size, and speed, the complex nature of web-based conversations requires innovative theory and methods, particularly to understand conversations in a specific context, such as in public health [8]. We lack a clear understanding of whether web-based conversations truly provide a channel for the exchange of different perspectives, whether people can communicate in an open and listening environment, and whether web-based conversations stimulate cognitive information processing.

To fill the gaps in the extant literature on web-based conversations, particularly in the health context, we proposed a 3-facet approach to understanding web-based health conversations: homophily of the conversational networks, relational dynamics between parties in the conversation, and cognitive information processing in the conversation. Homophily is the tendency of people to interact with others who share a preexisting characteristic or attitude. In our study, homophily is the tendency of people who share similar attitudes about vaccines to interact more frequently with each other than with people who hold differing views. Thus, first, homophily of the conversational networks on social media potentially inhibits the formation of bridging social capital, which is crucial for obtaining information from different perspectives and seeking advice on complex health-related issues [9,10]. Second, positive relational dynamics that are characterized by respect, openness, and connectedness [11] encourage a listening environment that fosters effective communication between parties with differing viewpoints. Finally, cognitive information processing stimulated by web-based conversations may foster issue-relevant knowledge building [6]. Specifically, by analyzing large-scale conversational text data on Reddit through deep neural network language models and computer-assisted conversational analyses, we examined individual conversational behaviors concerning a controversial public health issue (ie, vaccines) across three dimensions: (1) homophily, whether and how people selectively engage in conversations that are consistent with their attitudes; (2) language coordination, whether and how people accommodate their language when engaging in web-based conversations that oppose or align their attitudes, guided by the Communication Accommodation Theory (CAT); and (3) analytical thinking, whether and how people engage in motivated reasoning or analytical thinking when approaching opposing viewpoints, guided by dual-processing theories and motivated reasoning.

### Homophily in Web-Based Conversations About Vaccines

Technological affordances of social media have provided a myriad of messages and channels from which people may select. Decades of research have found that individuals tend to be consistent in their choices. Selectivity in a high-choice media environment and the tendency to remain consistent may lead to homophily; that is, people select attitude-congruent information, transmit attitude-consistent messages, and interact with like-minded individuals. Homophily is more likely to emerge as the intellectual specificity on which a community is built increases [12]. Reddit users typically engage in forums known as “subreddits,” which are self-selecting groups dedicated to topics of varying specificity. This makes Reddit a likely context for the proliferation of homophily.

Homophily may lead to an *echo chamber* effect, a closed system in which people tend to seek out and disseminate information that reinforces their preexisting beliefs, attitudes, and behaviors, which is typically devoid of counterattitudinal perspectives [13]. Empirical research on homophily provides a more focused perspective on how homophily works in specific settings and concerning particular topics [14], including health. People select health information that is congruent with their personal values and goals [15]. For example, using large-scale digital trace data, Johnson et al [16] found echo chambers of provaccination and antivaccine groups in which attractive narratives blend topics such as safety concerns and conspiracy theories. Del Vicario et al [17] found that selective exposure may fuel the formation of homogeneous clusters of Facebook users who believe in misinformation. Individuals find vaccine information that aligns with their preexisting beliefs to be more credible, useful, and convincing; nonexperts are more likely to choose to engage with material that confirms what they already thought, an effect that was more pronounced among individuals with higher levels of health literacy [18]. Thus, we expected to find homophily among Redditors who engaged in vaccine-related conversations. Therefore, we proposed the following hypothesis:

Hypothesis 1: Redditors are more likely to selectively reply to others who share similar stances on vaccines.

### Language Coordination in Web-Based Conversations About Vaccines

Although homophily leads us to expect that both pro- and antivaccine Redditors are more likely to interact with those who share their opinions (ie, in-group members), they also interact with those who hold opposing views (ie, out-group members). We define Redditors who connect the provaccine and antivaccine groups by replying to posts within both groups as *cultural bridges* [19]. Comparing the linguistic patterns of out-group conversations with in-group conversations offers a way to determine whether the relational dynamics between provaccine and antivaccine groups are positive. Positive relational dynamics in intergroup conversations may provide a favorable listening environment for information exchange [11]. We focused on 1 type of dynamics, *power dynamics*, through *language coordination* in web-based conversations, to examine conversations about vaccines on Reddit.

The CAT predicts and explains the adjustments people make to manage social interactions [20]. The constant change in one’s communicative behavior, either linguistic or other communicative symbols that people use to indicate their identities (eg, body language, hairstyles, and eating patterns), is called *accommodation*. Among different accommodative strategies, *convergence* is a specific accommodation strategy in which individuals adapt their communicative behaviors in linguistic (eg, speech rate and accents), paralinguistic (eg, utterance length), and nonverbal features (eg, gestures) to become more like the individual or group they interact with.

Convergence is a function of the social power between 2 parties [21] and generally takes 1 of 2 forms: *upward* or *downward* convergence. Upward convergence describes people in subordinate positions accommodating their communicative behaviors to people in superordinate positions; downward convergence reverses the direction of the flow. The power distance between people primarily originates from 2 sources [22,23]: status and dependence. Status-derived power distance originates from formal designations or informal reputations. Dependence-derived power distance arises when someone needs something from another person, which creates dependence on the second person and gives the second person temporal power over the first person. An example of dependence is when a person attempts to persuade someone who holds an opposing view. This creates a form of dependence on the target of persuasion, which then transforms into a power distance between the target and persuader. The CAT predicts that this may result in the persuader accommodating their language toward the target of the persuasion [23].

The CAT has been applied in the health domain. For instance, a laboratory experiment on accommodation behaviors in instant messaging conversations found a general tendency for convergence for both the length and duration of utterances about the human papillomavirus (HPV) vaccine [24]. Although convergence has been extensively studied in various domains, including health care, 3 limitations merit further study [21]. First, the specific ways in which people engage in accommodation remain unclear, such as the use of particular parts of language (eg, function words) or the use of communicative symbols (eg, gestures). Second, we have little understanding of the boundary conditions under which the accommodation of language and communicative symbols will occur. Third, the CAT has rarely been tested in social media, where the many-to-many mode of communication changes the nature of communication, which may change accommodation behaviors [25]. This study helps fill these gaps by examining the potential convergence of function words in social media conversations among people with different opinions about vaccine-related issues.

In this study, we examined *language coordination*, a specific form of accommodation, in conversations about vaccines on social media. Empirical studies have shown evidence of persuasive intent on both the provaccine and antivaccine sides. A study analyzing antivaccine websites found that antivaccine websites are persuasive because they mix scientifically proximate misinformation (eg, autism and brain injury) with value-based beliefs (eg, freedom and individuality) [26]. Provaccine campaigns typically aim to build public trust in experts and ensure that vaccinations reflect the best-available scientific knowledge, not political interests [27,28]. Thus, we expect both sides to try to persuade the other when they interact. This creates a form of dependence, which translates into power distance, resulting in the persuader accommodating their language to the target of persuasion [23]. Therefore, we predicted that language coordination will be higher in out-group conversations than in in-group conversations. Our second hypothesis is as follows:

Hypothesis 2: Redditors who post about vaccines accommodate their language more when interacting with out-group members who hold different views on vaccines than when interacting with in-group members.

### Analytic Thinking in Web-Based Conversations About Vaccines

Complex social and scientific controversies, such as the debate over vaccination, require critical thinking to evaluate scientific evidence and potentially reconsider one’s perspective [29]. However, research shows that when confronted with opposing views, people are likely to counterargue through directional motivated reasoning, reinforcing their preexisting beliefs [30-32]. Directional motivated reasoning refers to the phenomenon in which people have a directional goal in reasoning, such as a preferred interpretation [33]. Directional motivated reasoning is not a consequence of the overuse of heuristic processing but a result of highly cognitively involved systematic processing [34]. Systematic and heuristic processing are 2 routes of information processing according to dual-process models [35,36]. Systematic processing is a more cognitively demanding process during which individuals thoughtfully consider the information presented, whereas heuristic processing is a less cognitively demanding process during which simple cues in the messages influence attitudes [36]. We used *analytical thinking* to refer to the intentional and resource-demanding processing and *intuitive thinking* to refer to effortless and instinctive processing, following Epstein [37]. In addition to directional motivated reasoning, accuracy-driven reasoning may also occur when people encounter information inconsistent with their preexisting beliefs [32]. Individuals are more likely to make greater efforts in their analytic thinking when they lack sufficient confidence in the accuracy of their judgments (eg, encountering belief-incongruent information) [35]. Whether their resulting processing is directed or accuracy driven, we expect that people tend to engage in more resource-demanding analytical thinking compared with intuitive thinking when they encounter opposing views.

The flip side of this phenomenon can be seen when people encounter arguments that are consistent with their preexisting beliefs, which they are more likely to accept uncritically [31]; that is, people tend to use heuristics to make decisions when they are exposed to belief-congruent information because resource-demanding cognitive processing is typically avoided when people are confident about the accuracy of the information [38,39]. Therefore, analytical thinking will decrease when people encounter information that is consistent with their preexisting beliefs.

In the context of health, risk, and science communication, motivated reasoning potentially explains why people are susceptible to misinformation. Susceptibility to misinformation can ultimately be traced to people’s propensity to confirm previously held value-based beliefs and protect their social identities [40]. This goal can inspire motivated cognition related to policy-relevant facts [34]. For example, people motivated by equality and community believe that vaccinating teenage girls against HPV is essential to protecting women’s sexual health, whereas people who value hierarchy and individualism believe that universal vaccination against HPV among teenage girls will undermine young girls’ sexual health by increasing their chances of having unprotected sex [34]. Therefore, people are likely to engage in motivated reasoning when they encounter information that is inconsistent with their value-based beliefs about health and science, which is a result of intense analytical thinking.

Scholars have called for more research that incorporates cognitive models into vaccination education [41] and examines the role of belief systems using big data from social media platforms [42]. However, few studies have examined analytical thinking in the context of social media posts and comments. By analyzing 1489 comments on a Facebook post about childhood vaccination, Faasse et al [43] found that antivaccine comments typically showed greater analytical thinking. By analyzing 12,553 COVID-19 vaccine fact-checking posts and their comments on Facebook, Xue et al [44] showed that COVID-19 vaccine fact-checking posts continue to be more analytical over time. However, research has not yet investigated how analytical thinking changes when provaccine and antivaccine social media users interact with each other. On the basis of the theoretical work on motivated reasoning and dual processing as reviewed earlier, we proposed the third hypothesis regarding analytical thinking in conversations when provaccine and antivaccine Redditors interact with each other.

Hypothesis 3: Analytical thinking is higher when people interact with out-group members who hold opposing views than when they interact with in-group members who hold similar views.

## Methods

### Data Collection

We collected all posts and comments from the archive of Reddit hosted by Google’s BigQuery from January 1, 2016, to December 31, 2018. We then filtered the posts and their corresponding comments that are related to vaccines using a group of keywords (eg, vaccine, vacc, and vax; see Table S1 in Multimedia Appendix 1 [45-47] for a full list of the terms used). Next, we removed unrelated posts and comments, such as animal vaccines and vaccines used in video games (see the detailed removal criteria in the data collection section in Multimedia Appendix 1). We obtained 62,210 posts and 1,178,617 comments that met the aforementioned criteria, which served as the corpus for the following analyses.

### Classification Using Fine-tuned Pretrained Neural Network Language Models

We randomly selected 10.77% (6702/62,210) of the posts for expert coders to code into four categories of content using four dummy variables: (1) provaccine *message* if the post contains an idea that would be useful for someone who supports vaccinations or against vaccine hesitancy, (2) antivaccine *message* if the post contains an idea that would be useful to someone making an argument against vaccines, (3) provaccine *author* if we can tell the author of the post is provaccine, and (4) antivaccine *author* if we can tell the author of the post is antivaccine. We coded the message stance and the author stance separately because occasionally a post contains both provaccine and antivaccine information, but the author’s stance is clear despite the mixed information (see the detailed explanation in the measures section in Multimedia Appendix 1). Two expert coders independently coded all the randomly selected posts after training (see Table S2 in Multimedia Appendix 1 for intercoder reliability).

The coded posts served as training data for the 4 supervised machine learning models corresponding to the 4 categories. We used the pretrained language model in natural language processing, Decoding-Enhanced Bidirectional Encoder Representations From Transformers With Disentangled Attention (DeBERTa). BERT is a pretrained language model developed by Google. Specifically, for each category, we fine-tuned the 4 DeBERTa models with the same hyperparameters based on DeBERTaV3 [48] (see Table S3 in Multimedia Appendix 1 for the performance of the 4 models). The 4 fine-tuned DeBERTa models were used to predict the labels (ie, 0 or 1) of the 4 categories of the uncoded posts (n=55,508). The combination of the training set and machine-predicted posts served as the corpus of posts for further analyses (n=66,210). The comments that replied to the posts encompassed the corpus of comments for further analyses (n=1,178,617). Table 1 summarizes the distribution of posts and comments in terms of their stance on vaccines.

**Table 1:** The distribution of posts and comments in terms of their stance on vaccines.

	Posts (n=66,210), n (%)	Comments that reply to posts (n=1,178,617), n (%)^a^
Provaccine stance	23,680 (35.76)	653,154 (55.42)
Neutral^b^ stance	27,088 (40.91)	391,715 (33.24)
Antivaccine stance	9172 (13.85)	78,262 (6.64)
2-sided^c^ stance	2270 (3.43)	55,486 (4.71)
Total	66,210 (100)	1,178,617 (100)

^a^The stances are not the comments’ stance, but the posts’ stance to which the comments reply.

^b^Contains neither provaccine nor antivaccine information.

^c^Contains both provaccine and antivaccine information.

### Measures

*Post stance* was measured by integrating the values of 2 dummy variables: (1) provaccine message and (2) antivaccine message. If a post only contains provaccine information (ie, provaccine message=1 and antivaccine message=0), its post stance is a provaccine stance. Similarly, if a post only contains antivaccine information (ie, provaccine message=0 and antivaccine message=1), its post stance is an antivaccine stance. If a post contains both provaccine and antivaccine information (ie, provaccine message=1 and antivaccine message=1), its post stance is a 2-sided stance. If a post contains no opinions about vaccines or readers could not clearly identify its point of view (ie, provaccine message=0 and antivaccine message=0), its post stance is neutral (see the example posts of the 4 types of post stances in Table S4 in Multimedia Appendix 1).

*Author stance* was measured by comparing the number of times that an author of the post was predicted as provaccine or antivaccine. We predicted an author’s stance on vaccines every time they made a post related to vaccines. If an author has been predicted to be provaccine more times than antivaccine, then the author’s stance is provaccine. If an author has been predicted to be antivaccine more times than provaccine, then the author’s stance is antivaccine. If an author has been predicted as provaccine an equal number of times as antivaccine, then the author’s stance is mixed. If an author has been predicted to be neither provaccine nor antivaccine, then the author’s stance is unclear. We checked the prediction results of *provaccine author* and *antivaccine author* and found that no author made a large but equal number of provaccine and antivaccine posts but had 1 more case of 1 kind than the other. Therefore, the decision to use absolute numbers of provaccine and antivaccine posts is not sensitive to edge cases.

*Language coordination* between 2 speakers was measured by examining the similarity in language between the content of a comment and the content of the post or another comment that the comment is replying to. Specifically, for any pair of authors, *author a* and *author b*, *language coordination* was measured by the likelihood of *author b* using a specific linguistic style *marker m* in a comment *u_2_* that directly replies to a post or comment *u_1_*, which uses the same linguistic style *marker m* by *author a*. Language coordination measures how much *author a*’s use of *marker m* in a post or comment *u_1_* triggers the use of *marker m* by *author b* in a comment *u_2_* that directly replies to the post or comment *u_1_*, relative to *author b*’s normal use of *marker m* in conversations with *author a*. Given a set of conversations between *author a* and *author b* (*a*: *u_1_*, *b*:*u_2_*), we define the language coordination of *author b* toward *author a* as:



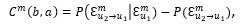



where 
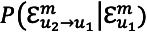
 defines the probability of *author b* uses *marker m* in the comment *u_2_* directly replying to *author a*’s post or comment *u_1_* that uses *marker m*, and 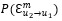 defines the probability of *author b*’s normal use of *marker m* in conversations with *author a*.

The linguistic style markers include 8 categories of function words generated by the Linguistic Inquiry and Word Count (LIWC) dictionary [49], including articles, auxiliary verbs, conjunctions, adverbs, impersonal pronouns, personal pronouns, prepositions, and quantifiers. We focused on function words rather than words that have substantive meanings to reduce the influence of topic-related content and to increase the generalizability of the findings of this study across different contexts [23]. In this study, we applied a generalized version of language coordination in which we measured a particular *author b* toward a group of *authors A* with the same stance on vaccines. Specifically, given a set of exchanges *S_A,b_* between *author b* and a group of authors *a* 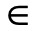 *A*. The set *S_A,b_* includes the posts or comments *u_1_* of various authors *a* 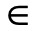 *A* and the comment *u_2_* of *author b*. The language coordination of *author b* to the group *A* is as follows:



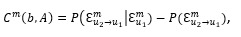



where the probabilities in the generalized equation are estimated over *S_A,b_*.

We used the coordination feature of the Python package *ConvoKit* (version 2.5.3) [50] to calculate each author’s language coordination toward the group of provaccine authors and the group of antivaccine authors. In other words, regardless of their own stance on vaccines, each author has 1 or 2 language coordination scores, depending on whether they replied to their in-group members, out-group members, or both.

*Analytical thinking* of each post or comment was measured by the analytical score using LIWC (see a detailed explanation of the measure and its validation in the measures section in Multimedia Appendix 1). The construct of analytical thinking has been defined as a deliberate mode of thought through which complex concepts are deconstructed into more manageable components and their interactions [45]. Language containing a high number of articles that typically signal concepts and prepositions that typically signal relationships between concepts is typically more analytical in nature because of the function of such linguistic devices [45]. Language containing a high number of pronouns, adverbs, negations, auxiliary verbs, and conjunctions typically indicates a more informal and personal style [45]. We focused on function words because they tend to be more reliable indicators of psychological states than content words with substantive meanings [46].

### Inferential Analysis

#### Homophily

To test whether Redditors with different opinions on vaccines are more likely to reply to their in-group members (hypothesis 1), we focused on authors who posted at least once about vaccines and left at least 1 comment about vaccines. (We did this for 2 reasons. Theoretically, we defined Redditors who both initiate and respond to posts related to vaccines as active participants in the public sphere on Reddit. Methodologically, we only coded the stances of post authors, not of the comment authors, due in part to the length of the text of comments often being too short for successful classification.) We then filtered the comment authors and posts whose stance is either provaccine or antivaccine to test hypothesis 1. We obtained 13,899 posts and 89,347 comments to use to test hypothesis 1. We then matched the corresponding posts’ stance to that of the authors of the comments that replied to the posts. Finally, we performed a chi-square test of independence to test the relationship between the 2 variables: *post stance* and comment *authors’ stance*.

#### Language Coordination

To test whether Redditors with different opinions on vaccines are more likely to coordinate language toward their in-group members or out-group members (hypothesis 2), we filtered posts and comments whose stance is either provaccine or antivaccine and whose count of function words is not equal to 0, as function words are integral to our measure of language coordination. We obtained 14,865 posts and 84,544 comments that met the criteria for further analyses. After obtaining the language coordination score(s) for each author depending on whether they replied to their in-group members, out-group members, or both, we then performed 2 separate 2-tailed independent sample *t* tests: one compared how provaccine and antivaccine Redditors coordinate their language toward antivaccine Redditors, and the other compared how provaccine and antivaccine Redditors coordinate their language toward provaccine Redditors. We further filtered out provaccine Redditors and antivaccine Redditors who replied to both their in-group members and out-group members (ie, cultural bridges) and performed 2-tailed paired-sample *t* tests to examine whether the patterns of linguistic styles differed for the same Redditor when they replied to in-group or out-group members.

#### Analytical Thinking

To test the interaction effect of post stance and comment authors’ stance on analytical thinking in the comments (ie, motivated reasoning; hypothesis 3), we filtered the posts and comments whose authors are either provaccine or antivaccine and whose count of function words is not equal to 0. Similar to our analysis of language coordination scores, we only used posts and comments with nonzero function words because analytical thinking was measured based on function words. We further selected the comments that directly replied to posts but not to other comments to avoid the confounding effects of other comments on the target comments. We obtained 7777 posts and 14,978 comments for further analysis. We then used multilevel modeling to control for the unmeasured interdependence between comments nested within the same authors, the same posts, or the same post authors (see the intraclass correlation coefficient of the intercept-only models in the inferential analysis section in Multimedia Appendix 1). The intraclass correlation coefficient showed a moderately high variability within the clusters (ie, same comment authors, posts, or post authors), so we considered the interdependence of the comments by applying a 3-level cross-classified model. We chose the 3-level cross-classified model because multiple comments could be separately affiliated with the same post and the same comment author (ie, cross-affiliated) and multiple posts could be affiliated with the same post author (ie, the third level). Therefore, the outcome variable (ie, analytical thinking in comments) was modeled at level 1. All predictors, including post stance, author stance, and post analytical thinking, were modeled at level 2. The equations are as follows:

Level 1: 



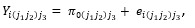



Level 2:



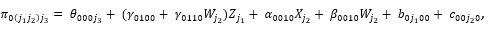



Level 3: 



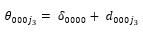



Combined: 



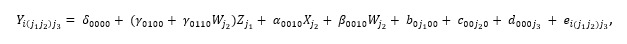



Where for each individual comment *i*, *j_1_*, *j_2_*, and *j_3_* represent the comment author, affiliated post, and post author, respectively; Z_j1_ represents the comment author’s stance, W_j2_ represents the post stance, and X_j2_ represents analytical thinking in the post; 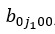, 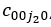, and 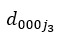 are random effects that vary randomly across comment authors, posts, and post authors, respectively.

### Ethical Considerations

This study used publicly available and accessible posts and comments from Reddit collected by the authors. Therefore, the activities described do not meet the requirements of human subject research and do not require review by an institutional review board.

## Results

Hypothesis 1, which is about whether provaccine and antivaccine Redditors selectively reply to their in-group members than to their out-group members, is supported. The results of the chi-square test of independence show that there was a significant relationship between post stance and comment authors’ stance (n*=*89,396; *χ*^2^_1_=42,210, *P*<.001). Both provaccine and antivaccine Redditors are more likely to reply to their in-group members than to their out-group members. Table 2 summarizes the cross-tabulation between post stance and comment authors’ stance.

**Figure 1:**
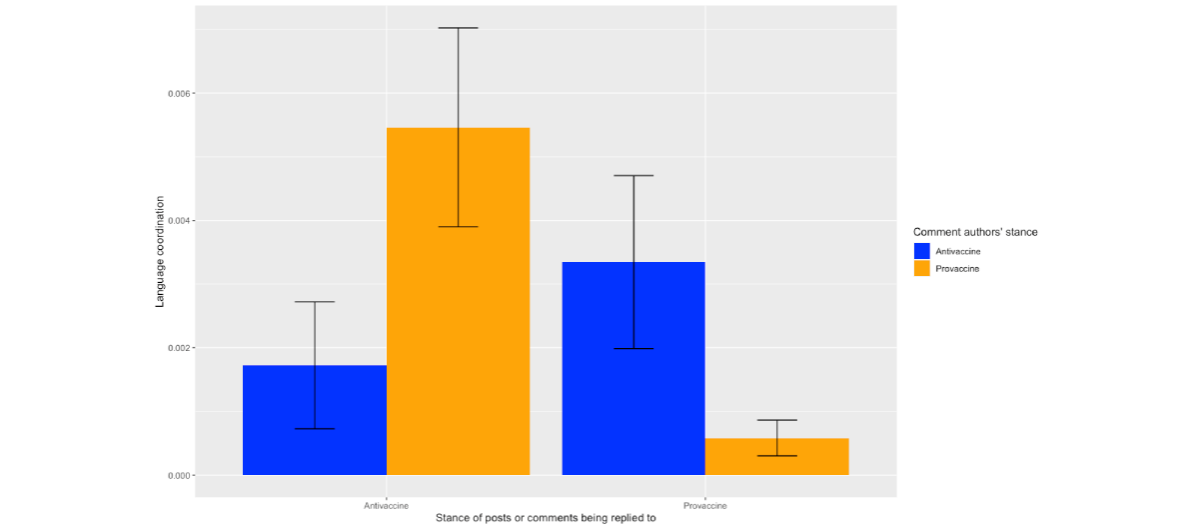
Interaction effects of the authors’ stances on language coordination.

Hypothesis 2, which is about whether provaccine and antivaccine Redditors are more likely to coordinate language toward out-group members than toward in-group members, is supported. Provaccine Redditors are more likely to coordinate their language toward antivaccine Redditors (mean 0.0055, SD 0.0351) than antivaccine Redditors coordinating their language toward antivaccine Redditors (mean 0.0017, SD 0.0195; t_819.35_=−2.02; *P*=.044). Antivaccine Redditors are more likely to coordinate their language toward provaccine Redditors (mean 0.0033, SD 0.0249) than provaccine Redditors coordinating their language toward provaccine Redditors (mean 0.0006, SD 0.0185; t_364.15_=1.99; *P*=.047). Figure 1 displays the interaction effects of authors’ stances on language coordination.

**Figure 2:**
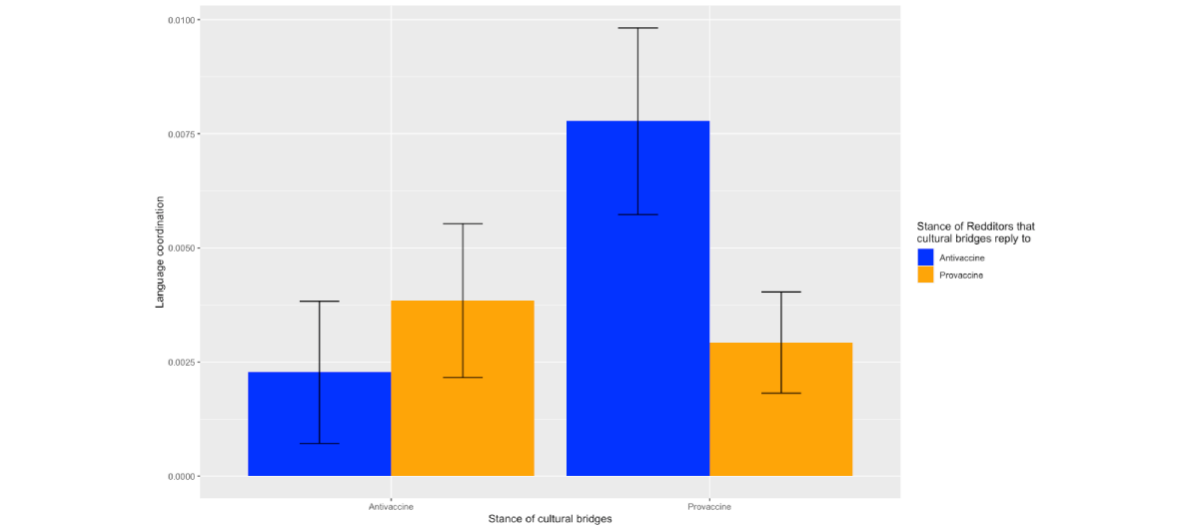
Cultural bridges’ differing language coordination toward in-group and out-group members.

For Redditors who serve as cultural bridges, antivaccine cultural bridges do not show significant differences between replying to provaccine Redditors (ie, out-group; mean 0.0038, SD 0.0232) and antivaccine Redditors (ie, in-group; mean 0.0023, SD 0.0215) in terms of language coordination. The difference in language coordination scores (0.0016, 95% CI −0.0029 to 0.0060) is not statistically significant (t_189_=0.70, *P*=.48). In contrast, provaccine cultural bridges tend to coordinate their language more toward antivaccine Redditors (ie, out-group; mean 0.0078, SD 0.0375) than toward provaccine Redditors (ie, in-group; mean 0.0029, SD 0.0203). The difference in language coordination scores (0.0048, 95% CI 0.0003-0.0094) is statistically significant (t_335_=2.08, *P*=.04). Figure 2 shows cultural bridges’ differing language coordination toward people who hold similar or dissimilar opinions on vaccines.

Hypothesis 3, which considers whether Redditors with different opinions on vaccines are more likely to engage in analytical thinking when they reply to out-group members, is not supported. Table 3 displays the results of the interaction between the post stance and comment author’s stance on analytical thinking in comments. On average, analytical thinking in the comments by provaccine authors was significantly lower than that in the comments by antivaccine authors (β=−12.87, SE 1.60, *P*<.001). Analytical thinking in comments replying to provaccine posts was significantly lower than that in comments replying to antivaccine posts (β=−2.89, SE 1.36, *P*=.03). We did not observe a significant interaction effect between post stance and comment author’s stance on analytical thinking in the comments (β=.89, SE 1.83, *P*=.63); that is, the difference in analytical thinking in the comments from provaccine Redditors and antivaccine Redditors does not depend on the stance of the posts that they reply to.

**Figure 3:**
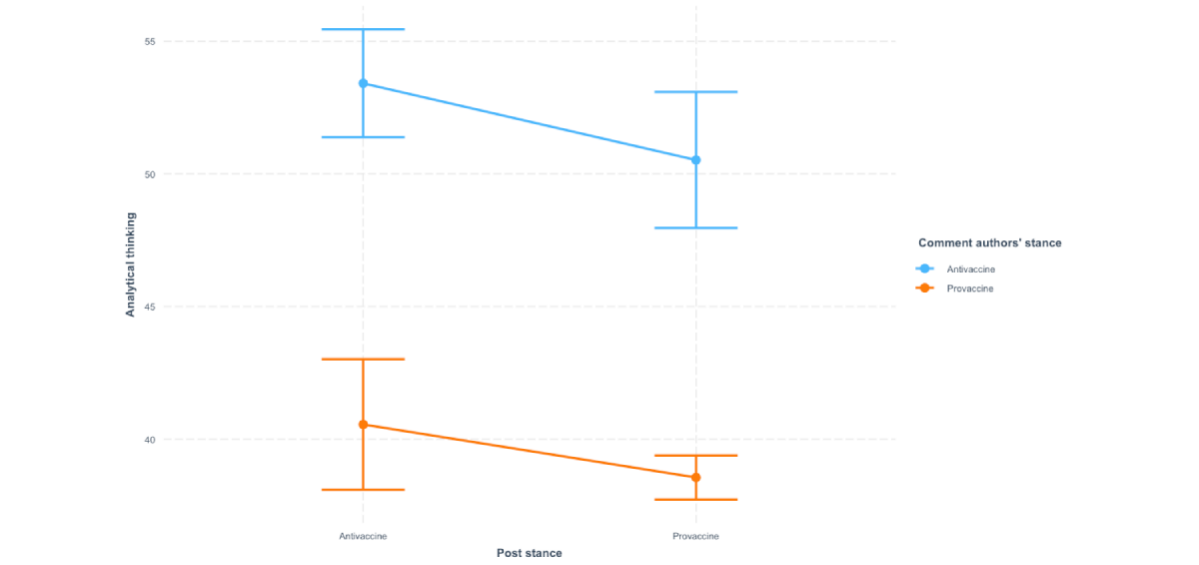
Interaction effects of post stance and comment author's stance on analytical thinking in comments.

**Figure 4:**
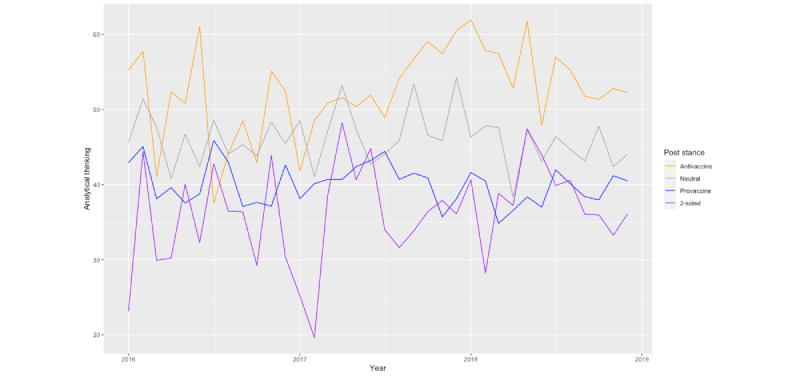
The trend of analytical thinking in comments grouped by the corresponding posts from 2016 to 2019.

**Figure 5:**
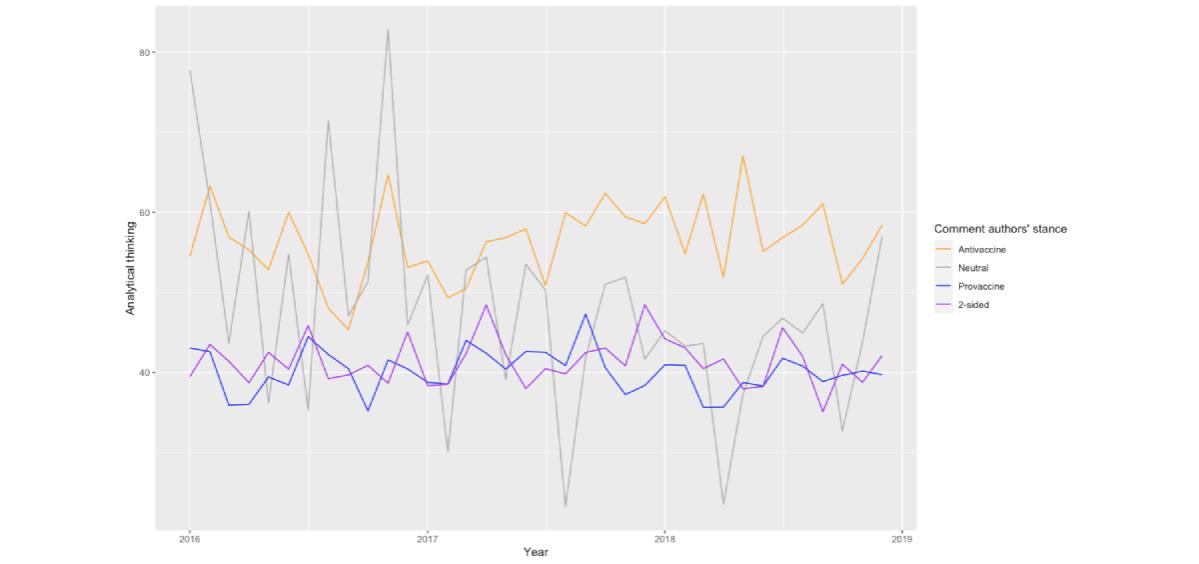
The trend of analytical thinking in comments grouped by the authors from 2016 to 2019.

Figure 3 shows the interaction effects between the post stance and the stance of the comment authors on analytical thinking in the comments. Regardless of the post stance, antivaccine Redditors’ comments, on average, involve more analytical thinking compared with provaccine Redditors’ comments. Similarly, regardless of the stance of the comment authors, the comments replying to antivaccine posts, on average, involve more analytical thinking than the comments replying to provaccine posts. We also visualized the trend of analytical thinking in the comments grouped by their posts’ stances and their authors’ stances in Figures 4 and 5. The trend from 2016 to 2018 is consistent with the aforementioned findings.

**Figure 6:**
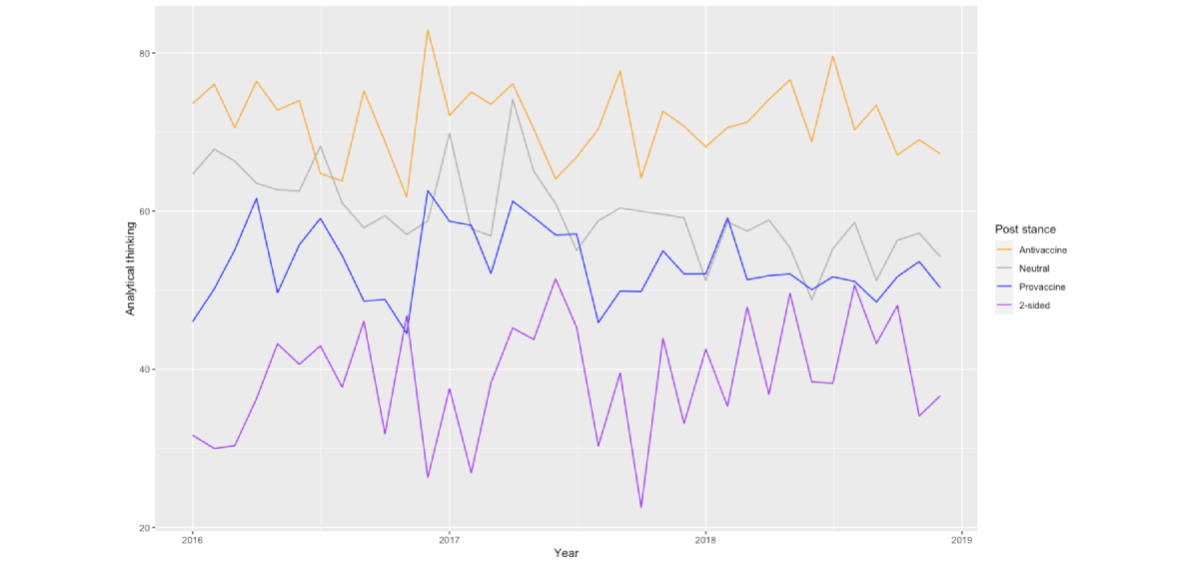
The trend of analytical thinking in posts about vaccines on Reddit from 2016 to 2019.

The results of the multilevel model also show that analytical thinking in the posts about vaccines is a significant predictor of analytical thinking in the comments. We visualized the trend of analytical thinking in the posts from 2016 to 2018 in Figure 6, which shows that antivaccine posts, on average, involve higher analytical thinking throughout the years than provaccine posts. This is consistent with the finding that analytical thinking in posts is a positive predictor of analytical thinking in comments.

**Table 2:** The cross-tabulation table between post stance and comment authors’ stance (n=89,396).

					Post stance							Total
					Provaccine			Antivaccine				
**Comment authors’ stance**												
	**Provaccine** **Redditors**											
		Values, n	63,811			4436			68,247			
		Within author stance (n=68,247; %)	93.5			6.5			100			
		Within post stance (provaccine: n=69,313; antivaccine: n=20,083; %)	92.1			22.1			N/A^a^			
		Total (n=89,396; %)	71.4			5			76.3			
	**Antivaccine Redditors**											
		Values, n	5502			15,647			21,149			
		Within author stance (n=21,149; %)	26			74			100			
		Within post stance (provaccine: n=69,313; antivaccine: n=20,083; %)	7.9			77.9			N/A			
		Total (n=89,396; %)	6.2			17.5			23.7			
**Total**												
	Values, n			69,313			20,083			89,396		
	Within author stance (%)			N/A			N/A			N/A		
	Within post stance (provaccine: n=69,313; antivaccine: n=20,083; %)			100			100			N/A		
	Total (n=89,396; %)			77.5			22.5			100		

^a^N/A: not applicable.

**Table 3:** The interaction effects of post stance and author stance on analytical thinking in comments.^a^

Fixed effects	Analytical thinking in comments		
	Estimated coefficients	SE	*P* value
Intercept	47.62	1.18	<.001
Comment author stance (pro)	–12.87	1.60	<.001
Post stance (pro)	–2.89	1.36	.03
Comment author stance × post stance	0.89	1.83	.63
Post analytical thinking	0.10	0.01	<.001

^a^The number of posts is 7777, and the number of comments is 14,985; the number of post authors is 5186, and the number of comment authors is 4366.

## Discussion

### Principal Findings

In this study, we examined the content and linguistic patterns of posts and comments about vaccines on Reddit. The results show that provaccine and antivaccine Redditors are more likely to selectively reply to Redditors who share similar views on vaccines. However, when Redditors interact with others who hold opposing views on vaccines, both provaccine and antivaccine Redditors accommodate their language to out-group members. However, for cultural bridges who interact with both in-group and out-group members, only provaccine cultural bridges accommodate their language toward out-group members. In addition, provaccine and antivaccine Redditors do not show greater analytical thinking when interacting with out-group members, suggesting that Redditors do not engage in motivated reasoning when interacting with people who hold different stances on vaccines. Antivaccine Redditors, on average, have higher analytical thinking in posts and comments than provaccine Redditors. These findings help further our understanding of web-based conversations in the public health domain and have implications for future provaccine campaign design on social media.

First, this study shows that both provaccine and antivaccine Redditors are more likely to comment on posts that share the same or similar views on vaccines. The results are consistent with previous research that shows that people are likely to seek health-related information on the internet that confirms prior beliefs [18] and that provaccine and antivaccine groups tend to form echo chambers [16]. The results indicate that provaccine and antivaccine users tend to form homogeneous communities that may inhibit the formation of bridging social capital. As bridging social capital is crucial for individuals being exposed to heterogeneous perspectives on a given issue (eg, vaccines) [10], our results imply that Redditors with different opinions on vaccines are less likely to exchange their arguments through Reddit. However, we underscore that we observe a certain number of Redditors who did bridge both sides by interacting with other Redditors who hold different opinions. This allowed us to further examine their language coordination and analytical thinking.

Second, our results show that provaccine and antivaccine Redditors accommodate their language when they interact with out-group members; that is, when both provaccine and antivaccine Redditors interact with those who hold opposing views, they tend to accommodate their linguistic styles accordingly, compared with the conversations with in-group members. This is a promising and optimistic finding, as listening to one another is the first step in any kind of effective communication. This indicates that Redditors with different opinions on vaccines display positive and healthy relational dynamics, which provides an open and connected environment to exchange arguments. However, when we filter cultural bridges who communicate with both in-group and out-group members, the results show a slightly different story. Compared with communication with in-group members, only provaccine cultural bridges accommodate language use when communicating with out-group members. This is consistent with what the CAT predicts would occur when people attempt to convince out-group members, which creates a power distance between those with opposing views [20,23], and with empirical studies that have found that persuading the public to build trust in experts and science is the major goal of provaccine campaigns [27,28]. This implies that when communicating with others who hold opposing views, provaccine cultural bridges are more open and proactive, whereas antivaccine cultural bridges do not show similar openness. This reluctance among antivaccine cultural bridges may pose an obstacle to effective vaccination campaigns, as they play a crucial role in changing the attitudes, perceptions, and behaviors of antivaccine users. Public health practitioners who use social media–based campaigns may want to design tailored interventions that target the language use of antivaccine cultural bridges.

Third, the results of analytical thinking in posts and comments do not support our hypothesis that provaccine and antivaccine Redditors engage in motivated reasoning when they interact with others who hold different opinions about vaccines. These findings are partially consistent with the findings of Pennycook and Rand [51], who found that susceptibility to misinformation is not because of motivated reasoning but because of a lack of reasoning using laboratory experiments. Although we found that antivaccine Redditors may not engage in motivated reasoning, which is consistent with Pennycook and Rand [51], our findings indicate that antivaccine Redditors have relatively high analytical thinking rather than a lack of reasoning regardless of whom they interact with, which is inconsistent with Pennycook and Rand [51]. One potential reason for the inconsistency could be that Pennycook and Rand [51] conducted laboratory experiments, whereas this study used large-scale social media data. The different levels of validity and generalizability of the 2 approaches may have contributed to the differences in the findings. Future studies may want to use different methods to further explore whether people who fall into health- or science-related misinformation engage in motivated reasoning and the extent of their analytical thinking.

It is important to note that the antivaccine Redditors’ comments and the comments replying to antivaccine posts, on average, involve more analytical thinking than their provaccine counterparts. In other words, web-based conversations about vaccines consistently stimulated more cognitive information processing among vaccine opponents than among vaccine proponents from 2016 to 2018. These findings are consistent with those of Faasse et al [43], who found that antivaccine comments typically showed higher analytical thinking than provaccine comments on Facebook. One reason that vaccine opponents show analytical linguistic styles in their posts and comments on social media could be that vaccine opponents tend to incorporate science-like language in their discourse with logically constructed statements and with fewer emotional expressions [52]. However, the psychological drivers of analytical thinking of vaccine opponents and the specific content of their analytical thinking are unclear. Future studies should further examine the content of antivaccine posts and comments on social media to answer these questions.

### Theoretical and Practical Contributions

This study contributes to the theoretical understanding of web-based conversations between people with opposing views through the lens of homophily, the CAT, and motivated reasoning. The 3 theories help deepen our understanding of web-based conversations between people with opposing views on controversial public health issues, as discussed earlier. This study also extends the CAT by testing whether people accommodate a particular type of communicative symbol (ie, function words) on social media, in which the many-to-many mode of communication changes the nature of communication. In addition, this study extends the literature on motivated reasoning by testing it in a real-world setting using social media data.

This study has several important practical implications. First, the findings deepen our understanding of how vaccine proponents and opponents interact on social media. Although previous research has explored this at the macro level, there is a lack of deep understanding of micro level interactions between vaccine proponents and opponents. Our study fills this gap through the lens of language coordination and analytic thinking in posts and comments on vaccines on social media. Our findings provide an optimistic view that vaccine proponents and opponents accommodate their language and do not engage in motivated reasoning when interacting with people with opposing views. Public health practitioners can build on our research by designing campaigns that use social media to facilitate communication and mutual understanding between the 2 sides.

Second, the findings offer potential explanations for why some social media–based provaccine campaigns do not achieve the expected effect and may have implications for the future design of strategic campaigns. We found that when communicating with others who hold opposing views on vaccines, provaccine cultural bridges accommodate language use, whereas antivaccine cultural bridges do not. This indicates that although antivaccine cultural bridges play a crucial role in the success of provaccine campaigns, they are not as proactive as they could be, as reflected by their lack of language accommodation. Future social media–based provaccine campaigns may want to target these cultural bridges to facilitate communication between the 2 sides. In addition, our findings suggest that provaccine posts have not successfully stimulated analytical thinking on either side. Low analytical thinking may hinder the understanding of information in provaccine campaigns. Future social media–based provaccine campaigns may want to design messages and corresponding visuals to increase analytical thinking among audiences. Third, we found that provaccine cultural bridges and general vaccine proponents accommodate their languages when interacting with vaccine opponents on social media. Future provaccine campaigns may include moderators or bots to proactively send provaccine messages in languages accommodated toward vaccine opponents.

### Limitations and Future Directions

This study has several limitations that future research should examine. First, this study relies on a single item from the LIWC dictionary to measure analytical thinking. Although previous studies have used the analytical thinking score from the LIWC to answer various research questions [45-47], the reliability and validity of the measure in the context of social media are less clear. Future studies should use different ways to operationalize analytical thinking in conversations on social media to examine whether the findings still hold. Second, the sampling strategy used in this study may have led to bias. This study only included authors who have posted at least 1 post on Reddit to test hypothesis 1 to hypothesis 3 for both theoretical and methodological considerations. Therefore, Redditors who only commented on the posts but had not posted posts themselves were excluded from the sample. In addition, we only used first-level comments (ie, comments that directly replied to the posts) to test hypothesis 3 considering the potential confounders if including all the comments. These sampling strategies may introduce selection bias in the sample. Future studies should examine whether the findings hold when all comments and Redditors are included. Third, data were collected between 2016 and 2018 before the COVID-19 pandemic. The COVID-19 pandemic has changed web-based discussions about vaccines and people’s perceptions and attitudes toward vaccines. Future studies should explore whether these findings can be generalized to the postpandemic era. Fourth, this study did not differentiate between different types of vaccines; nuances of attitudes toward vaccines; and characteristics of Redditors that may be related to vaccine attitudes, such as gender, nationality, and partisan identity, among others. Future studies are encouraged to explore how different audience characteristics, such as gender and nationality, may affect attitudes toward vaccines and how social media users with more nuanced attitudes interact with each other. Fifth, although this study did not find differences in the effects of different subreddits on the patterns of conversations (Table S5, Multimedia Appendix 1), future studies may explore how subreddits with different structures and affordances affect user participation patterns.

### Conclusions

This study examined the content and linguistic patterns of conversations about vaccines in Reddit from 2016 to 2018 using deep learning and computer-assisted conversational analyses. This study found that although people are more likely to reply to posts that share similar views, people accommodate their language styles and do not engage in motivated reasoning when they interact with people with opposing views. Theoretically, this study deepens our understanding of the nature of web-based conversations, particularly web-based conversations about vaccines. Practically, this study has implications for future social media–based provaccine campaign design, such as increasing analytic thinking in campaign messages and targeting cultural bridges to facilitate conversations between those who support and oppose vaccination.

## Appendix

Multimedia Appendix 1 Supplemental materials for the methods, measures, and inferential analyses.

## Abbreviations

CAT: Communication Accommodation Theory
DeBERTa: Decoding-Enhanced Bidirectional Encoder Representations From Transformers With Disentangled Attention
HPV: human papillomavirus
LIWC: Linguistic Inquiry and Word Count

## Data Availability

The data sets generated and analyzed during this study are available in the Harvard Dataverse repository [53].
