# Supplementary material for: Examining Homophily, Language Coordination, and Analytical Thinking in Web-Based Conversations About Vaccines on Reddit: Study Using Deep Neural Network Language Models and Computer-Assisted Conversational Analyses
Source: J Med Internet Res. 2023 Mar 23;25:e41882. doi: 10.2196/41882 (PMC10131607; doi:10.2196/41882)
Supplement: Multimedia Appendix 1 [file jmir_v25i1e41882_app1.docx]

**Supplementary Information for**

How do vaccine proponents and opponents interact with each other on social media? Examining selective exposure, language coordination, and analytical thinking in online conversations about vaccines.

**This WORD file includes:**

**Supplementary text**

Methods

Data collection

Classification using supervised machine learning

Measures

Language coordination

Validation of analytical thinking via PCA

Inferential analysis

Analytical thinking

Additional analysis with subreddit as a control variable

**Figures S1 to S4**

S1: Variables plot of PCA for analytical thinking score using posts

S2: Variables plot of PCA for analytical thinking score using comments

S3: Scree plot of PCA for analytical thinking score using posts

S4: Scree plot of PCA for analytical thinking score using comments

**Tables S1 to S3**

S1: List of keywords used to filter out vaccine-related posts

S2: Intercoder reliability between two coders for the four categories

S3: Performance of the four machine learning models

S4: Example posts in the four categories

S5: Additional analysis with subreddit as a random effect term

S6: Additional analysis on homophily in top 2 subreddits

**SI References**

Methods

**Data collection.** We removed the posts and comments if (1) they are about animals’ vaccines, (2) they show evidence of having been posted by bots (i.e., auto summaries of news articles, user names containing “bot”, subreddits designated for bots), (3) they discussed vaccines used in video games rather than real vaccines (e.g., “vaccines” are used in the video game “Digimon” to prevent important data from being destructed by Virus Digimon), (4) they are non-English, (5) the word count of the post is less than four (in which case many of our measures of language use are likely to not function correctly), and (6) the percentage of words containing “vacc” or its variants (i.e., vax) in the post is less than 0.002 (these tend to be long posts that mention vaccination in passing and are about other topics).

**Classification using supervised machine learning.** In most cases (95%) in which we were able to code both the stance of the author and the message the two were congruent, but in a small subset (5%) they did not match. For example, a real post on Reddit reads, “my husband and mom are completely against it [Tdap vaccine] and say, ‘it can cause malformities in babies’ or ‘babies don’t need it, you don’t need it’. We get into arguments about it anytime it’s brought up … I just want my baby girl to get the most protection she can get.” This post contains both pro-vaccine and anti-vaccine information, but we can tell the author is pro-vaccine.

Measures

**Language coordination.** For any pair of authors, *author a* and *author b*, *language coordination* was measured by the likelihood of *author b* using a specific linguistic style *marker m* in a comment *u_2_* that directly replies to a post/comment *u_1_*, that uses the same linguistic style marker *m* by *author a*. Language coordination measures how much *author a*’s use of *marker m* in a post/comment *u_1_* triggers the use of *marker m* by *author b* in a comment *u_2_* that directly replies to the post/comment *u_1_*, relative to *author b*’s normal usage of *marker* *m* in conversations with *author a*. Given a set of conversations between *author a* and *author b* (*a*: *u_1_*, *b*:*u_2_*), we define the language coordination of *author b* toward *author a* as:

$C^{m}\left( b,a \right)=P\left( ℇ_{u_{2}\to u_{1}}^{m} \right|ℇ_{u_{1}}^{m})-P(ℇ_{u_{2}\to u_{1}}^{m})$,

where $P\left( ℇ_{u_{2}\to u_{1}}^{m} \right|ℇ_{u_{1}}^{m})$ defines the probability of *author b* uses *marker m* in the comment *u_2_* directly replying to *author a*’s post/comment *u_1_* that uses *marker m*, and $P(ℇ_{u_{2}\to u_{1}}^{m})$ defines the probability of *author* *b*’s normal usage of *marker m* in conversations with *author* *a*.

In the current study, we applied a generalized version of language coordination, in which we measured a particular *author b* towards a group of *authors A* with the same stance on vaccines. Specifically, given a set of exchanges *S_A,b_* between *author b* and a group of authors *a* ∈ *A*. The set *S_A,b_* includes the posts/comments *u_1_* of various authors *a* ∈ *A* and the comment *u_2_* of *author* *b*. The language coordination of *author b* to the group *A* is:

$C^{m}\left( b,A \right)=P\left( ℇ_{u_{2}\to u_{1}}^{m} \right|ℇ_{u_{1}}^{m})-P(ℇ_{u_{2}\to u_{1}}^{m})$,

where the probabilities in the generalized equation are estimated over *S_A,b_*.

**Validation of analytical thinking via Principal Component Analysis.** The analytical thinking variable in “LIWC is generated by a factor analysis of the function word categories… with articles and prepositions being positively loaded and the remaining six dimensions being negatively loaded” (Jordan et al., 2019). Previous work has validated the use of the analytical thinking variable using college admission essays (Pennebaker et al., 2014), literature of Theobald, Shakespeare, and Fletcher (Boyd and Pennebaker, 2015), and speeches of political leaders (Jordan et al., 2019). We have validated the analytical thinking score by performing the principal component analysis (PCA) on both posts and comments using the function words generated by LIWC, following previous studies (Pennebaker et al., 2014). For both posts and comments, the analytical thinking scores from LIWC and the PCA are highly correlated (*r_posts_* = 0.93; *r_comments_* = 0.91). We validated the analytical thinking score by performing the principal component analysis (PCA) on both posts and comments using the function words generated by Linguistic Inquiry and Word Count (LIWC), following the steps in the previous studies ^1–3^. We first generated eight categories of function words using LIWC: personal pronouns (e.g., I, his, we), impersonal pronouns (it, thing), auxiliary verbs (is, have), articles (an, the), prepositions (to, on), conjunctions (and, or), negations (no, never), and common adverbs (really, very). We then performed a PCA on the eight categories of function words. As shown in Fig. S3 and Fig. S4, there is only one principal component in the conversational text using both posts and comments. Fig. S1 and Fig. S2 indicate that the principal component accounts for 27.7% of the variance of function words used in the posts and 22.6% of the variance of function words used in the comments. Fig. S1 and Fig. S2 also show that articles and prepositions are in the same direction and the remaining six categories are in the same direction when projecting on the principal component. The results are generally consistent with all the previous studies that articles and prepositions reveal analytical thinking in which complex concepts are deconstructed into manageable components and their relationships ^1^. The remaining six categories, such as pronouns, adverbs, and auxiliary verbs reflect a more informal and personal language style ^1^.

It is important to note that there is an adjustment we applied to the standard process of performing PCA on function words. We removed all the posts and comments that contain no function words from the sample. It is because the results would be severely biased towards these zero-function-words cases to explain their variances based on the algorithms of PCA. The previous studies did not need to perform the pre-processing because they typically used long texts. We used social media posts/comments data, which are typically short due to the nature of social media. Therefore, we added a step of pre-processing to the standard PCA procedures based on the previous studies.

Inferential analysis

**Analytical thinking.** We used multilevel modeling to control for the unmeasured interdependence between comments that are nested within the same authors, the same posts, or the same post authors. From the intercept-only models where intercepts are allowed to vary across posts, post authors, and comment authors, respectively, the intraclass correlation coefficient (ICC) (i.e., the correlation between the analytical thinking scores of the comments from the same clusters) is 0.181 for comments from the same authors, 0.126 for comments from the same posts, and 0.114 for comments from the same post authors.

**Additional analysis.** To further test whether subreddits will affect analytical thinking in the comments, we added a random effect term of subreddit to examine whether Reddit users have different conversational patterns in different subreddits. The results show that conversational patterns between Redditors with different attitudes toward vaccines do not vary across different subreddits. The results table is in Table S5.

To further test whether homophily in conversations about vaccines differs in different subreddits, we ran chi-square tests of independence to test the relationship between post stance and comment authors’ stance in the top 2 subreddits where Redditors discuss vaccines the most. The results are consistent with the finding in all posts and comments about vaccines. That is, there was a significant relationship between post stance and comment authors’ stance, *X^2^* (1, N = 26049) = 19861, *P* <.001. Both pro-vaccine and anti-vaccine Redditors are more likely to comment on posts that share the same or similar views on vaccines in the top 2 subreddits: conspiracy and vaxxhappened. Table S6 summarizes the crosstabulation between post stance and comment authors’ stance in the top 2 subreddits.

Fig. S1. Variables plot of PCA for analytical thinking score using posts


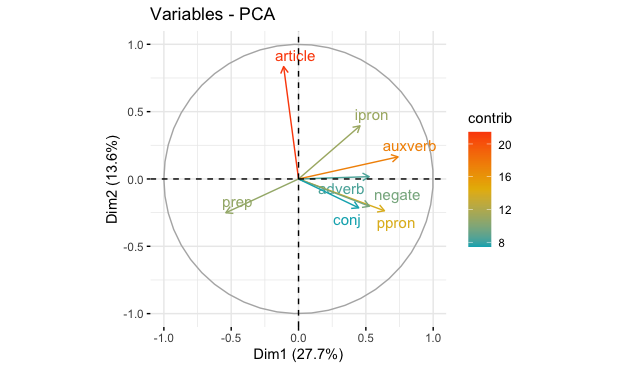


Fig. S2. Variables plot of PCA for analytical thinking score using comments


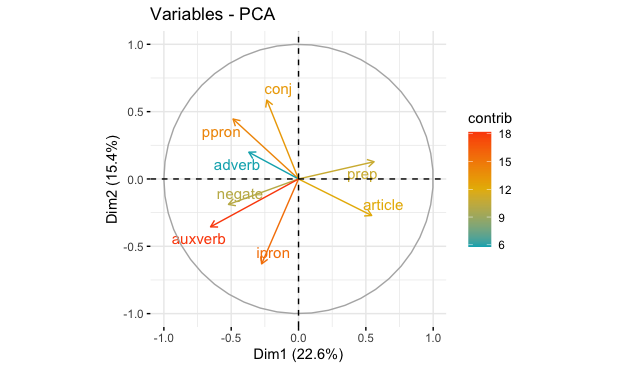


**Fig. S3.** Scree plot of PCA for analytical thinking score using posts


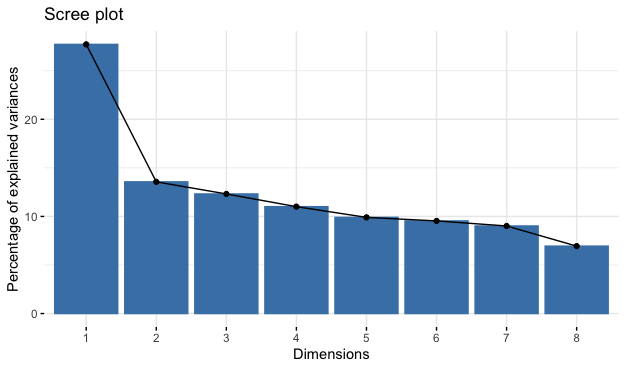


**Fig. S4.** Scree plot of PCA for analytical thinking score using comments


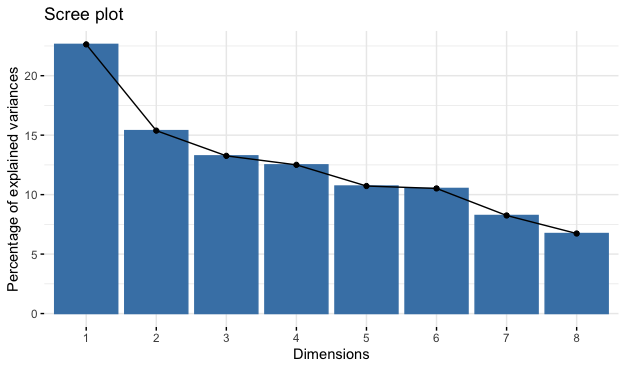


**Table S1.** *List of keywords used to filter out vaccine-related posts*

| **No.** | **Key words** |
| --- | --- |
| 1 | vaccine |
| 2 | vaccinat |
| 3 | vaccs |
| 4 | vaccer |
| 5 | anti-vax |
| 6 | pro-vax |
| 7 | antivax |
| 8 | provax |
| 9 | anti vax |
| 10 | pro vax |
| 11 | vaxer |
| 12 | vaxxer |
| 13 | vaxine |
| 14 | vaxxine |
| 15 | vaxed |
| 16 | vaxxed |
| 17 | vaxing |
| 18 | vaxxing |

**Table S2.** *Intercoder reliability between two coders for the four categories*

|  | pro-vaccine message | anti-vaccine message | pro-vaccine author | anti-vaccine author |
| --- | --- | --- | --- | --- |
| Krippendorff’s alpha | 0.76 | 0.77 | 0.75 | 0.84 |

**Table S3.** *Performance of the four machine learning models*

|  | accuracy | macro average F1 | weighted average F1 |
| --- | --- | --- | --- |
| message pro-vaccine | 0.84 | 0.83 | 0.84 |
| message anti-vaccine | 0.92 | 0.88 | 0.92 |
| author pro-vaccine | 0.83 | 0.82 | 0.83 |
| author anti-vaccine | 0.94 | 0.87 | 0.94 |

Note: F1-score = 2*Recall*Precision/(Recall+Precision)

Precision = True Positive/(True Positive + False Positive)

Recall = True Positive/(True Positive + False Negative)

**Table S4.** *Example posts in the four categories*

| **Category** | **Example posts** |
| --- | --- |
| pro-vaccine | **Ex 1**: MMR vaccines not linked to Autism, we're in luck.  **Ex 2**: people complaining about vegans telling them what to eat are like anti-vaxxers complaining about being told how to raise their children. It's no longer a personal choice once it causes unnecessary pain, suffering and death to other beings. |
| anti-vaccine | **Ex 1**: Mercury in vaccines causes cellular mitochondria to suffocate and die.  **Ex 2**: If vaccines are safe, why has the US gov. paid out $3 BILLION to vaccine-injured families? |
| neutral | **Ex 1**: WHO expects to set link between Zika and microcephaly in weeks, vaccine 18 months.  **Ex 2**: Hey guys, background - traveling to Suzhou, China (90miles from Shanghai) on a business trip. CDC recommends Typhoid and Hep. A vaccines for travelers to China. I'm male, 25, Caucasian, roughly 6' and 165lbs with no known allergies or health problems. Should I get vaccinated? |
| two-sided | **Ex 1**: My husband and mom are completely against it [Tdap vaccine] and say, ‘it can cause malformities in babies’ or ‘babies don’t need it, you don’t need it’. We get into arguments about it anytime it’s brought up … I just want my baby girl to get the most protection she can get.  **Ex 2**: Before you lecture me or accuse me of being an anti-vaxxer...my baby started having epileptic seizures after her 4 month shots. She's on seizure meds twice a day ever since. It sucks to not know whether vaccinating again would be safe. It also sucks to be at risk for the diseases. Ugh. I hate this. |

**Table S5.** *Additional analysis with subreddit as a random effect term*

|  | | Analytical Thinking in Comments | | | |
| --- | --- | --- | --- | --- | --- |
| Fixed Effects | | Estimated Coefficients | | Standard Errors | P value |
| Intercept | | 50.25*** | | 1.61 | <.001 |
| Comment author stance (pro) | | -12.55*** | | 1.79 | <.001 |
| Post stance (pro) | | -4.04** | | 1.65 | .008 |
| Comment author stance × Post stance | | 2.24 | | 1.96 | .25 |
| Post analytical thinking | | 0.08*** | | 0.01 | <.001 |
| Random Effects | |  |  | |  |
| Groups | Name | Variance | Standard Deviation | |  |
| Post | Intercept | 15.87 | 3.98 | | - |
| Comment author | Intercept | 117.39 | 10.83 | | - |
| Post author | Intercept | 7.81 | 2.79 | | - |
| Subreddit | Intercept | 29.61 | 5.44 | |  |
| Residual |  | 878.23 | 29.64 | | - |
| Number of comments | | 14985 | | |  |
| Number of posts | | 7777 | | |  |
| Number of comment authors | | 4366 | | |  |
| Number of post authors | | 5186 | | |  |
| Number of subreddits | | 770 | | |  |

*Note: *** P <.001, ** P <.01, * P <.05*

**Table S6.** *Additional analysis on homophily in top 2 subreddits* (*N = 26049*)

|  | | | Post stance | | Total |
| --- | --- | --- | --- | --- | --- |
|  |  |  | Pro-vaccine | Anti-vaccine |  |
| Comments author stance | Pro-vaccine | Count | 12388 | 1303 | 13691 |
|  |  | % within author stance | 90.5% | 9.5% | 100% |
|  |  | % within post stance | 97.0% | 9.8% | -- |
|  |  | % of total | 47.6% | 5.0% | 52.6% |
|  | Anti-vaccine | Count | 378 | 11980 | 12358 |
|  |  | % within author stance | 3.1% | 96.9% | 100% |
|  |  | % within post stance | 3.0% | 90.2% | -- |
|  |  | % of total | 1.5% | 46.0% | 47.4% |
| Total |  | Count | 12766 | 13283 | 26049 |
|  |  | % within author stance | -- | -- | -- |
|  |  | % within post stance | 100% | 100% | -- |
|  |  | % of total | 49.0% | 51.0% | 100% |

**SI References**

1. Jordan KN, Sterling J, Pennebaker JW, Boyd RL. Examining long-term trends in politics and culture through language of political leaders and cultural institutions. *Proc Natl Acad Sci*. 2019;116(9):3476-3481. doi:10.1073/pnas.1811987116

2. Pennebaker JW, Chung CK, Frazee J, Lavergne GM, Beaver DI. When Small Words Foretell Academic Success: The Case of College Admissions Essays. *PLOS ONE*. 2014;9(12):e115844. doi:10.1371/journal.pone.0115844

3. Boyd RL, Pennebaker JW. Did Shakespeare Write Double Falsehood? Identifying Individuals by Creating Psychological Signatures With Text Analysis. *Psychol Sci*. 2015;26(5):570-582. doi:10.1177/0956797614566658
